# Human mesenchymal stem cells target adhesion molecules and receptors involved in T cell extravasation

**DOI:** 10.1186/s13287-015-0222-y

**Published:** 2015-12-10

**Authors:** Federica Benvenuto, Adriana Voci, Enrico Carminati, Francesca Gualandi, Gianluigi Mancardi, Antonio Uccelli, Laura Vergani

**Affiliations:** Department of Neurology, Rehabilitation, Ophthalmology, Genetics, Maternal and Child Health (DINOGMI), University of Genoa, IRCCS-AUO San Martino-IST, Largo Paolo Daneo 3, 16132 Genova, Italy; Centre of Excellence for Biomedical Research (CEBR), University of Genoa, Viale Benedetto XV 7, 16132 Genova, Italy; Department of Earth, Environment and Life Sciences (DISTAV), University of Genoa, Corso Europa 26, 16132 Genova, Italy; Division of Hematology and Bone Marrow Transplant Unit, IRCCS-AUO San Martino-IST, Largo Rosanna Benzi 10, 16132 Genova, Italy

**Keywords:** mesenchymal stem cells, CD3^+^-selected lymphocytes, human endothelial cells, immunosuppressive effects, surface adhesion molecules and receptors, transendothelial migration, leukocyte migratory potential

## Abstract

**Introduction:**

Systemic delivery of bone marrow-derived mesenchymal stem cells (MSC) seems to be of benefit in the treatment of multiple sclerosis (MS), an autoimmune disease of the central nervous system (CNS) sustained by migration of T cells across the brain blood barrier (BBB) and subsequent induction of inflammatory lesions into CNS. MSC have been found to modulate several effector functions of T cells. In this study, we investigated the effects of MSC on adhesion molecules and receptors on T cell surface that sustain their transendothelial migration.

**Methods:**

We used different co-culture methods combined with real-time PCR and flow cytometry to evaluate the expression both at the mRNA and at the plasma-membrane level of α4 integrin, β2 integrin, ICAM-1 and CXCR3. In parallel, we assessed if MSC are able to modulate expression of adhesion molecules on the endothelial cells that interact with T cells during their transendothelial migration.

**Results:**

Our *in vitro* analyses revealed that MSC: *(i)* inhibit proliferation and activation of both peripheral blood mononuclear cells (PBMC) and CD3^+^-selected lymphocytes through the release of soluble factors; *(ii)* exert suppressive effects on those surface molecules highly expressed by activated lymphocytes and involved in transendothelial migration; *(iii)* inhibit CXCL10-driven chemotaxis of CD3^+^ cells; *(iv)* down-regulated expression of adhesion molecules on endothelial cells.

**Conclusions:**

Taken together, these data demonstrate that the immunosuppressive effect of MSC does not exclusively depends on their anti-proliferative activity on T cells, but also on the impairment of leukocyte migratory potential through the inhibition of the adhesion molecules and receptors that are responsible for T cell trafficking across BBB. This could suggest a new mechanism through which MSC modulate T cell responses.

## Introduction

The central nervous system (CNS) is an immune-privileged site where the endothelial blood–brain barrier (BBB) tightly controls lymphocyte entry. Under physiological conditions, lymphocyte traffic across the BBB is low, but during inflammatory diseases of the CNS—such as multiple sclerosis (MS) or its animal model, experimental autoimmune encephalomyelitis (EAE)—circulating CD4^+^ and CD8^+^ lymphocytes gain access to the CNS leading to inflammation, demyelination, and neurodegeneration [1–3]. Preventing the migration of lymphocytes into the CNS seems to be an important therapeutic approach for MS, and adhesion molecules on lymphocytes have long been studied as possible targets [4, 5] possibly leading to the translation into relevant therapies for MS [6].

Integrins are one of the major families of adhesion molecules that mediate binding of leukocytes to vascular endothelium leading to their extravasation [7], and they are probably involved in demyelinating diseases [8–10]. Integrins typically consist of α and β subunits [7]; α4 integrin interacts with β1 integrin forming the very late antigen-4 (VLA-4) antigen expressed on T and B cells. VLA-4 is responsible for lymphocyte binding to endothelial cells [8, 9]; the ability of T cells to induce EAE seems to correlate with α4 integrin expression [10, 11]. In addition, the monoclonal antibody (mAb) natalizumab (NTZ), developed to treat relapsing–remitting MS, selectively binds α4 integrin on autoreactive T cells and thus reduces their extravasation into CNS [12]. Also β2 integrin acts in leukocyte trafficking as a constituent of the lymphocyte function-associated antigen-1 (LFA-1) that binds the intercellular adhesion molecules (ICAMs) on endothelial cells [3]. Other molecules seem to be involved in the pathogenesis of MS. In particular, ICAM-1, a member of the Immunoglobulin Superfamily (IgSF) that is weakly expressed on resting lymphocytes but highly expressed on endothelial cells [13, 14], is induced by inflammatory mediators [14–16] and acts in transmigration of T cells, of T-helper type (Th) 17 lymphocytes in particular [17]. Also the chemokine receptor CXCR3 on the lymphocyte surface seems to act in the patrolling of the CNS [18] and may play a role in MS pathogenesis [19] because it triggers rapid leukocyte adhesion and is induced in activated CD4^+^ and CD8^+^ T cells crossing the BBB [18–20].

The molecular counterparts for lymphocyte trafficking into the CNS are the adhesion molecules on endothelial cells of the BBB, whose expression typically increases under inflammatory conditions [21]. Among these, the most important are ICAM-1 and the activated leucocyte cell adhesion molecule (ALCAM) that are upregulated by inflammatory stimuli [22, 23]. In particular, postmortem sections from patients with MS and mice with EAE showed higher expression of ALCAM. Moreover, mAbs against ALCAM reduced the accumulation of CD4^+^ T cells, decreased the disease severity, and delayed the onset of EAE [22, 23]. For this reason, ALCAM has been recently associated with risk, development, and progression of MS [24].

Mesenchymal stem cells (MSC) are progenitor cells of mesodermal origin that can be found in almost every connective tissue, including the bone marrow where they have been well characterized [25]. MSC are capable of modulating immune responses [26, 27] by having an anti-apoptotic effect on different cell types including T lymphocytes [28] and neurons [29, 30], as well as by releasing trophic factors and favoring endogenous neurogenesis [29–31]. MSC have therefore emerged as a promising tool for therapeutic applications in neurological experimental conditions, such as for the treatment of EAE, through inducing T-cell tolerance against myelin antigens, reducing T-cell infiltrates in the CNS [32], inhibiting the encephalitogenic potential of autoreactive T cells and axonal loss [33], and protecting from oxidative stress damage [31, 34]. Since the 1990s, MSC have been used in clinical settings in humans to promote the engraftment of hematopoietic stem cells (HSC) [35, 36] and to treat immune-mediated diseases such as acute graft versus host disease (GvHD) [37]. Some studies demonstrated that MSC inhibit cell proliferation and decrease interferon gamma (IFNγ) production in T cells, probably through the secretion of soluble factors but also by “cell to cell contact” mechanisms [28, 38, 39]. In addition, soluble proinflammatory factors released by activated lymphocytes strongly influence MSC modulatory activity, supporting the idea of an intense cross-talk between MSC and immune cells [40, 41]. Despite the large amount of studies, little is known on the ability of MSC to affect the expression of molecules sustaining the lymphocyte–BBB interaction necessary for the lymphocytes to gain access to inflamed tissues.

Here we hypothesized that MSC may modulate the capability of activated lymphocytes to cross the BBB and reach the CNS, at least in part, by selectively acting on specific adhesion molecules and chemokine receptors involved in the extravasation of activated T cells. In the light of their possible implication in pathogenesis of MS, we explored the ability of human MSC to modulate the expression, both at the mRNA level and at the plasma-membrane level, of α4 integrin, β2 integrin, CXCR3, and ICAM-1 on CD3^+^ lymphocytes, and of ICAM-1 and ALCAM on endothelial cells.

## Methods

### Chemicals

All chemicals, unless otherwise indicated, were of analytical grade and were obtained from Sigma-Aldrich Corp. (Milan, Italy).

### Human mesenchymal stem cell isolation and cell culture

Human bone marrow samples were obtained from healthy donors undergoing bone marrow explant from allogeneic transplantation procedures. Informed consent, approved by the local Ethics Committee (Regione Liguria), was obtained from all donors. Donors’ age ranged between 22 and 54 years (mean = 38.6). Mononuclear cells were isolated by density gradient centrifugation (1.077 g/ml, Lympholyte Cell separation Media; Cedarlane Laboratories Ltd, Burlington, Ontario, Canada), and cultured in Human Mesencult Basal Medium additioned with its specific supplement (Stem Cell Technologies, Vancouver, BC, Canada) at 37 °C in a humidified 5 % CO_2_ atmosphere_._ MSC growing as adherent cells were harvested at 80 % of confluence using Trypsin 0.05 %–ethylenediamine tetraacetic acid (EDTA) 0.02 % and then expanded. MSC were characterized by flow cytometry at each passage and used upon phenotypic characterization as CD73^+^, CD44^+^, CD105^+^, CD90^+^, CD45^−^, CD34^−^, and CD14^−^ cells [28]. For each experiment we used the BM-derived MSC from a single donor, and the experiment was repeated using MSC from different donors.

### Peripheral blood mononuclear cell isolation, CD3^+^ lymphocyte selection, and coculture with MSC

Peripheral blood mononuclear cells (PBMC) were separated by density gradient centrifugation from heparinized blood of healthy donors after informed consent [28]. PBMC were resuspended in RPMI 1640 supplemented with l-glutamine, penicillin, streptomycin and 10 % fetal calf serum (FCS) and dispensed in microtiter 200 μl plates (Sarstedt S.r.l, Verona, Italy) at a concentration of 1 × 10^5^ cells/well. Upon stimulation with 2 μg/ml anti-Human OKT3 (R&D Systems, Milan, Italy) and 1 μg/ml anti-Human CD28 (BD Biosciences, San Jose, CA, USA) antibodies, PBMC were cultured alone or in the presence of MSC from different donors at three different PBMC/MSC ratios (20:1, 10:1, and 4:1). After 4 days, cells were harvested and used for experiments.

CD3^+^ lymphocytes were isolated from the PBMC by magnetic negative selection using a Pan T isolation kit (Miltenyi Biotech GmBh, Bergish Gladbach, Germany). Briefly, PBMC were resuspendend in RPMI without FCS, plated at a concentration of 2 × 10^6^ cells/ml, and maintained for 5 minutes at 37 °C to allow the adhesion of monocytes. The monocyte-depleted cells were then harvested, resuspended in phosphate-buffered saline (PBS)–0.02 % EDTA at a concentration of 10^7^ cells/40 μl, and incubated with 10 μl Biotin Antibody Cocktail for 10 minutes at 4 °C. After incubation with anti-Biotin microbeads for 15 minutes, cells were washed, resuspended in PBS–0.02 % EDTA (10^7^ cells/500 μl), and applied on MS columns (Miltenyi Biotech GmBh) following the manufacturers’ instructions. CD3^+^ cells were then employed in transwell experiments using flat-bottom microtiter plates (Millipore, Milan, Italy). CD3^+^ cells were dispensed at a concentration of 1 × 10^5^cells/well in the lower chamber previously coated with αCD3 (10 μg/ml) and αCD28 (1 μg/ml) mAbs. MSC were seeded in the upper chamber and three different CD3^+^/MSC ratios were used (20:1, 10:1, and 4:1). After 4 days, cells were harvested and used for experiments.

For all treatments we checked the viability of cells by Trypan Blue staining, which resulted to be over 95 % also at the highest coculture ration with MSC ratio (4:1).

### Human endothelial cell culture and expansion

Human endothelial cells (HECV; Cell Bank and Culture in GMP-IST, Genoa, Italy) were maintained at 37 °C in a humidified 5 % CO_2_ atmosphere in Dulbecco’s modified Eagle’s medium high glucose (DMEM) supplemented with l-glutamine, penicillin, streptomycin and 10 % FCS. HECV were characterized by flow cytometry as CD166^+^, CD105^+^, CD54^+^, CD146^+^ MHC class I, CD102^−^, and CD106^−^ cells. In coculture experiments, HECV were dispensed at a concentration of 1 × 10^6^ cells/well using flat-bottom 24-well plates and stimulated with recombinant human IFNγ (200 IU/ml; Invitrogen Co., Carlsbad, California, USA). Cells were then incubated with MSC from different donors in transwell conditions (TW) at three different HECV/MSC ratios (20:1, 10:1, and 4:1) for 24 hours. MSC were seeded in the upper chamber and HECV in the lower chamber. Before seeding HECV were irradiated at 5000 rad, washed once with PBS, counted, and plated with or without MSC.

### RNA isolation and quantitative real-time PCR

Total RNA was isolated from either lymphocytes or endothelial cells using the Trizol reagent (Sigma Aldrich, Saint Louis, Missouri, USA) [42]. First-strand cDNA was synthesized in a Master-cycler apparatus (Eppendorf, Milan, Italy) from 1 μg total RNA as described elsewhere [43]. Gene expression levels were quantified by quantitative real-time RT-PCR (qPCR) using Chromo4TMSystem PCR apparatus and iTaq SYBR Green Supermix (Biorad, Milan, Italy). Amplification reactions were performed in a final volume of 25 μl containing 0.3 μM each primer, 10 ng cDNA, 1× SybrGreen PCR Master Mix. All primers (Table 1) were designed *ad hoc* starting from the coding sequences available on the GenBank database (http://www.ncbi.nlm.nih.gov/Genbank/GenbankSearch.html) and were synthesized by TibMolBiol custom oligosynthesis service. A melting curve of RT-PCR products (55–94 °C) was acquired to ensure the absence of artifacts. Relative expression of target mRNA was calculated using the comparative Cq method and was normalized for the expression of *GAPDH* gene [44]. The normalized expression was thus expressed as the relative quantity of mRNA (fold induction) with respect to controls (C).

**Table 1:** Sequences of the primer pairs used for quantitative real-time RT-PCR analysis

Gene	Accession number	Forward primer	Reverse primer
		5' → 3'	5' → 3'
α4 integrin	NM_000885.4	GGAATATC AGTTTTTACACAAAGG	AGA GAG CCA GTC CAG TAA GAT GA
β2 integrin	NM_001114380.1	GCTGTCCCCACAAAAAGTG	CCG GAA GGT CAC GTT GAA
CXCR3	NM_001142797.1	CCAGCCATGGTCCTTGAG	GGG CCG TAC TTC CTC AAC T
ICAM-1	NM_000201.2	CAACCGGAAGGTGTATGAAC	CGA GGT GTT CTC AAA CAG CTC
IFNγ	NM_000619.2	GGCATTTTGAAGAATTGGAAAG	TTT GGA TGC TCT GGT CAT CTT
ALCAM	NM_001627.2	CGT CTG CTC TTC TGC CTC TT	TAG GTG CCT CAA ACA CGT TG
GAPDH	NM_002046.3	AACCACTCCTCCACCTTTGACGC	CTC TTG TGC TCT TGC TGG GGC TG

*ALCAM* activated leucocyte cell adhesion molecule, *IFNγ* interferon gamma, *ICAM* intercellular adhesion molecule

### Flow cytometric analysis of lymphocyte surface antigens

Cells were stained with the specific primary mAb for 30 minutes at 4 °C, washed once with PBS, and analyzed. For coculture experiments, cells were additionally stained with Live Dead Fixable Near–IR Dead Cell-Stain Kit (Invitrogen) for 30 minutes at room temperature to exclude apoptotic cells by flow cytometric gating strategies (FSC-A vs. FL6-A dotplot). All immunolabeling procedures, unless otherwise indicated, were performed in the dark. The following mAbs were employed: CD34FITC, CD73PE, CD44FITC, CD14FITC, CD45FITC, CD45PE-Cy5, CD54APC, CD54PE-Cy5 (BD Biosciences), CXCR3FITC and CXCR3APC (R&D Systems), CD49d PE, CD90PE-Cy5, CD105APC, CD102PE, and CD106 APC (Biolegend Europe BV, London, UK), and KI67FITC (Dako Italia SpA, Milan, Italy). On the CD3^+^ lymphocyte population, the proportion of cells expressing α4 integrin, ICAM-1, and CXCR3 in the different experimental conditions was measured. On HECV, we recorded the shift in the mean fluorescence intensity (MFI) for each adhesion molecule under the different experimental conditions. Moreover, production of IFNγ by activated CD3^+^ lymphocytes was determined using Flow Cytomix particle-based assay (Biosciences, Prodotti Gianni, Milan, Italy), according to the manufacterer’s instructions [45]. All flow cytometric analyses were performed by a FACS Canto flow cytometer (BD Biosciences) and data were collected and analyzed by DIVA software (BD Biosciences). Flow Cytomix particle-based assay data were analyzed with FlowCytomixPro 1.0 Software, eBioscience, San Diego, California, USA.

### CD3^+^ cell proliferation analysis

Cell proliferation was measured by ^3^H-thymidine (^3^H-TdR) incorporation. CD3^+^ cells cultured in the absence or in the presence of MSC in a transwell system were pulsed with 0.5 μCi/well ^3^H-TdR (5 Ci/mmole specific activity; GE Healthcare Europe GmbH, Milan, Italy) for 8 hours. At the end of incubation, cells were harvested onto Multiscreen Harvest plates (Millipore, Billerica, MA, USA) using a 96-well plate-automated cell harvester (Tomtec, Handem, CT, USA). Scintillation liquid (Fisher Chemicals, Leicester, UK) was then added and ^3^H-TdR incorporation was measured by liquid scintillation spectroscopy using a beta-counter (Chameleon TM 425-104 Multilabel Counter -Bioscan, Washington, USA). The results expressed in counts per minute (kcpm, cpm × 1000) are given as the mean value of triplicate wells. In the same experiments, CD3^+^ cells cocultured as already described were also analyzed by flow cytometry for Ki67 intranuclear expression to identify KI67^+^ cycling T cells.

### CD3^+^ lymphocyte migration analysis

Chemotaxis of CD3^+^ lymphocytes was investigated using 24-transwell plates with 5 μm pore size polycarbonate membrane (Corning Costar, Celbio, Milan, Italy) as reported elsewhere [46]. CD3^+^ lymphocytes were grown for 48 hours with or without MSC (4:1 ratio) in the presence of αCD3 (10 μg/ml) and αCD28 (1 μg/ml) in a transwell system. Then, 5 × 10^5^ CD3^+^ lymphocytes were dispensed in the upper chamber, whereas 600 ng/ml CXCL10 (IP10; R&D Systems) or medium alone was added to the lower chamber. Plates were incubated for 2 hours at 37 °C, and then cells that migrated into the lower chamber were harvested and counted. Results were expressed as % input, calculated as the % ratio between the number of CD3^+^ cells dispensed in the upper chamber and that of cells recovered from the lower chamber after migration. Net % input (namely the difference between the input obtained following chemokine stimulation and that obtained with medium alone) was used for statistical analysis of the results.

### Statistical analysis

Data of qPCR are expressed as mean ± standard deviation (SD) of two independent RNA extractions from at least five independent experiments, and each PCR was performed in quadruplicate. Data of flow cytometry are expressed as mean ± SD of the frequency of populations measured in at least five independent experiments. Statistical significance was determined by analysis of variance followed by the Bonferroni post-hoc test (Instat software; GraphPad Software, Inc., San Diego, CA, USA). The Mann–Whitney *U* test was used for chemotaxis-related experiments.

## Results

### MSC inhibit T-cell proliferation and function

**Fig. 1:**
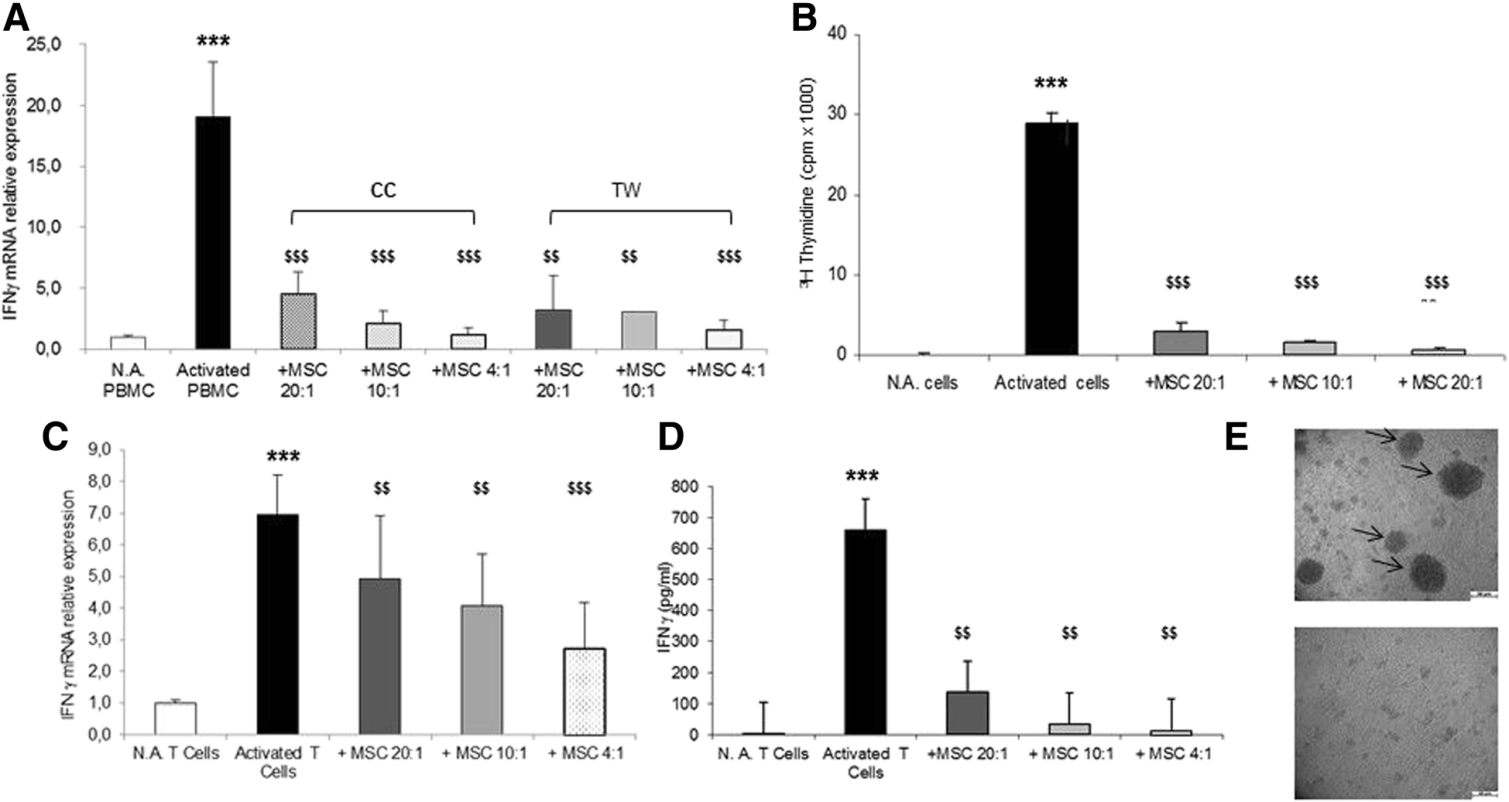
Effects of MSC on T-cell proliferation and function. In PBMC in resting conditions (*N.A.*) or after activation via αCD3/αCD28 mAb, we assessed the mRNA expression of IFNγ by qPCR **a** and cell proliferation by ^3^H-TdR incorporation **b** in the absence or in the presence of MSC for all PBMC/MSC ratios (20:1, 10:1, and 4:1), either in CC (*squared bars*) or in TW (*solid colored bars*) condition. In activated CD3^+^ lymphocytes we assessed both the mRNA expression **c** and secretion **d** of IFNγ in the absence or in the presence of MSC at all CD3/MSC ratios (20:1, 10:1, and 4:1) in TW. Cluster formation of CD3^+^ lymphocytes was assessed by light microscopy **e**. Images were acquired after 2-day stimulation of CD3^+^ lymphocytes with plate-bound αCD3/αCD28 in the absence or in the presence of MSC (CD3/MSC 4:1 ratio) in TW (20× magnification). Clusters are marked with *arrows*. PCR data (mean ± SD) are reported as fold induction with respect to controls after normalization for GAPDH mRNA. Significant differences are denoted by symbols on bars: activated vs. N.A. cells, *p ≤0.01, **p ≤0.001, ***p ≤0.0001; MSC-treated vs. MSC-untreated cells, $*p* ≤0.01, $$*p* ≤0.001, $$$*p* ≤0.0001. *IFNγ* interferon, *MSC* mesenchymal stem cells, *PBMC* peripheral blood mononuclear cells

In preliminary experiments, activated PBMC were cocultured with MSC at different PBMC/MSC ratios (20:1, 10:1, and 4:1) using both the cell-to-cell contact (CC) and TW in order to select the experimental model to be used in the study. When IFNγ expression was assessed by qPCR, a significant upregulation was observed in activated PBMC with respect to controls (19-fold; *p* <0.001) (Fig. 1a). In the CC condition (Fig. 1a), MSC led to a dose-dependent reduction in IFNγ expression with a decrease of −94 % with respect to activated cells (*p* <0.001) in the presence of the highest MSC dose (PBMC/MSC ratio 4:1), and of about −80 % (*p* <0.001) for the other ratios. Similar effects were observed in TW (Fig. 1a), where the maximum reduction in IFNγ mRNA level (−92 % with respect to activated cells; *p* <0.001) was observed at the 4:1 PBMC/MSC ratio, but a significant decrease of about −83 % (*p* <0.01) was observed also at 20:1 and 10:1 ratios. As these data clearly indicate that MSC effects on PBMC do not depend on CC, we used TW to test the possible effects of MSC on PBMC proliferation by using ^3^H-TdR incorporation (Fig. 1b). PBMC consistently proliferated upon *in vitro* activation (mean 0.11 ± 0.03 vs. 29.02 ± 1.31, unstimulated vs. stimulated, respectively; *p* <0.001). A dose-dependent inhibition of cell proliferation was observed when cells were cocultured with MSC at all PBMC/MSC ratios for 4 days (means 3.05 ± 1.07, 1.68 ± 0.14, and 0.67 ± 0.18 for 20:1, 10:1, and 4:1 ratios, respectively; *p* <0.001). Correspondingly, most PBMC cultured in the absence of MSC constitutively expressed Ki67, a classical marker of cell proliferation, and its expression strongly decreased in the presence of MSC in a dose-dependent manner (data not shown).

Using TW, expression and secretion of IFNγ was measured in selected CD3^+^ lymphocytes cocultured with MSC in TW (Fig. 1c). After activation, CD3^+^ cells showed a marked upregulation of IFNγ expression (about sevenfold with respect to control; *p* <0.001) that was significantly reduced in the presence of MSC. The maximum reduction (−67 % with respect to activated cells; *p* <0.001) was observed at the highest MSC dose (CD3/MSC ratio 4:1), but a significant decrease of about −33 % (*p* <0.01) was observed also at 20:1 and 10:1 ratios (Fig. 1c). On the same samples, the secretion of IFNγ in the medium was evaluated using flow cytometry (Fig. 1d). The IFNγ level increased in activated CD3^+^ cells (141-fold with respect to resting cells; *p* <0.001) and decreased in a dose-dependent manner when CD3^+^ cells were cocultured with MSC at all CD3/MSC ratios (*p* <0.01) (Fig. 1c).

Finally, the ability of CD3^+^ cells to form cluster was assessed by light optical microscopy (Fig. 1e, upper and lower). According to expectations, CD3^+^ cell clusters were observed upon stimulation with αCD3/αCD28 (Fig. 1e, upper), while the presence of MSC in coculture with CD3^+^ cells prevented cluster formation (Fig. 1e, lower).

### MSC inhibit expression of adhesion molecules on CD3^+^ lymphocytes

At first, the basal expression of the adhesion molecules under analysis was assessed in resting CD3^+^ lymphocytes. While α4 integrin mRNA was expressed in basal conditions, both ICAM-1 and β2 integrin were barely detectable (Cq values of about 25 for α4 integrin, and 32 for ICAM-1 and β2 integrin) (data not shown).

**Fig. 2:**
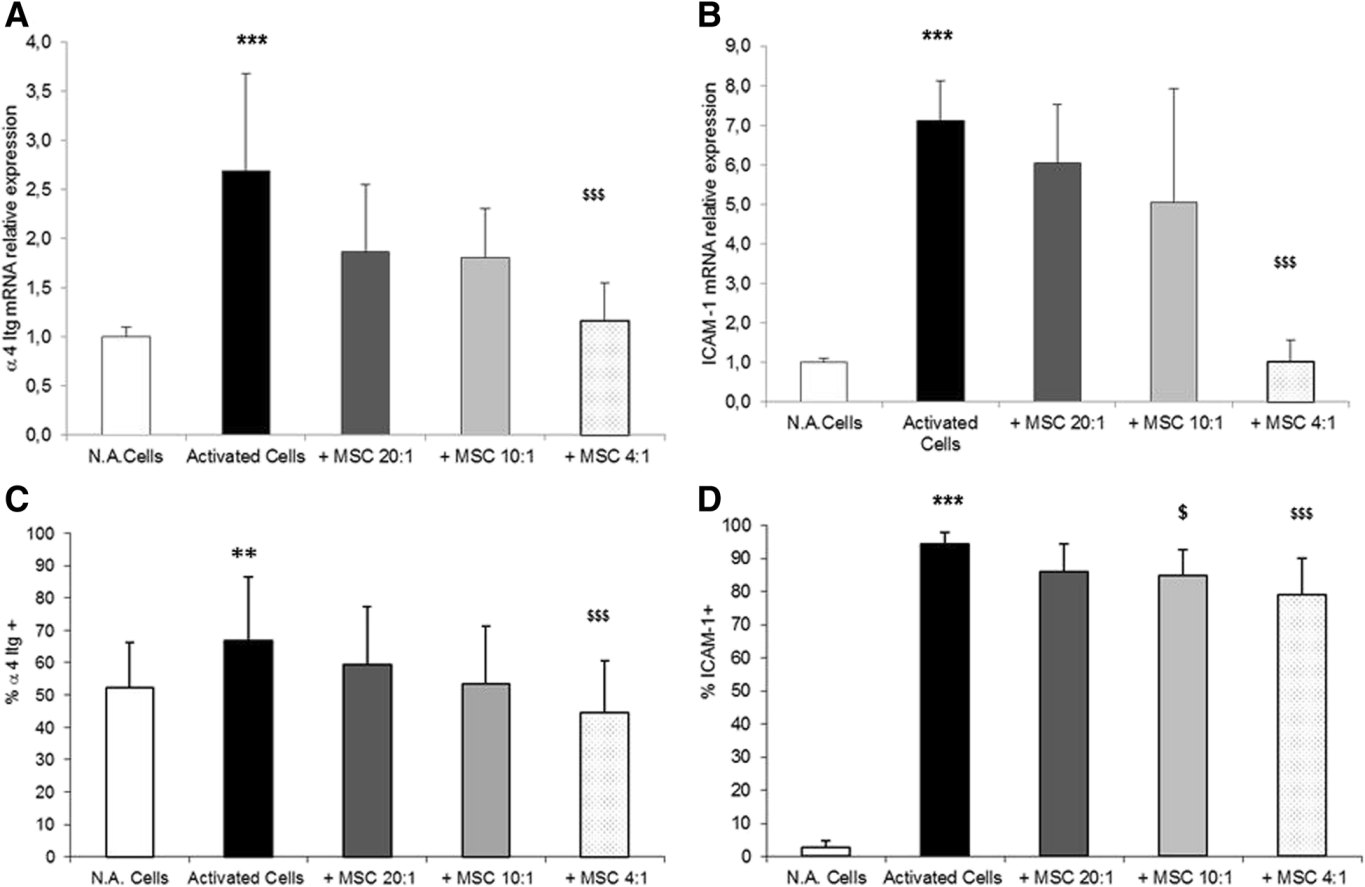
Effects of MSC on the expression of CD3^+^ lymphocyte adhesion molecules. In activated CD3^+^ lymphocytes cultured in the absence or in the presence of MSC in TW, the relative mRNA expression of α4 integrin **a** and of ICAM-1 **b** was evaluated by qPCR. Data (mean ± SD) are reported as fold induction with respect to controls after normalization for GAPDH mRNA. The proportion of cells which express α4 integrin **c** and of ICAM-1 **d** on the T-cell membrane was evaluated by flow cytometry in the same experimental conditions. Results are expressed as mean percentage ± SD obtained from six independent experiments. Activated vs. N.A. cells, *p ≤0.01, **p ≤0.001, ***p ≤0.0001; MSC-treated vs. MSC-untreated cells, $*p* ≤ 0.01, $$*p* ≤0.001, $$$*p* ≤0.0001. *ICAM* intercellular adhesion molecule, *MSC* mesenchymal stem cells, *N.A.* PBMC in resting conditions

At mRNA level, α4 integrin expression was upregulated upon activation with respect to resting CD3^+^ lymphocytes (2.7-fold induction; *p* <0.001) (Fig. 2a). A significant reduction with respect to activated cells (−57 %; *p* <0.001) was observed with the highest MSC dose in TW (4:1 CD3/MSC ratio); an evident but not significant effect could be appreciated also at 20:1 and 10:1 ratios. Activated CD3^+^ cells showed also a significant increase in mRNA expression of ICAM-1 with respect to resting lymphocytes (7.1-fold induction; *p* <0.001) (Fig. 2b). Also in this case the highest MSC dose significantly reduced ICAM-1 expression with respect to activated CD3^+^ cells (−86 %; *p* <0.001) (Fig. 2b). For comparison, similar experiments were performed on PBMC cocultured with MSC at different PBMC/MSC ratios (20:1, 10:1, and 4:1) using both CC and TW, and similar inhibitory effects were observed in both culture conditions (data not shown).

Although activated CD3^+^ cells did not show any significant increment in mRNA level of β2 integrin with respect to resting cells, a significant reduction of about −67 % (*p* <0.001) was observed for all the CD3/MSC ratios without a dose dependence (Table 2).

**Table 2:** Relative expression of β2 integrin mRNA in activated CD3^+^ lymphocytes cultured in the absence or in the presence of MSC at three CD3/MSC ratios (20:1, 10:1, and 4:1)

Treatments	Fold induction
Activated CD3^+^ cells	0.87 ± 0.28
Activated CD3^+^ cells + MSC 20:1	0.29 ± 0.33**
Activated CD3^+^ cells + MSC 10:1	0.30 ± 0.35**
Activated CD3^+^ cells + MSC 4:1	0.28 ± 0.18***

mRNA expression was evaluated by qPCR, and values representing the mean ± SD of three different experiments are reported as fold induction with respect to controls (no activated CD3^+^ lymphocytes) after normalization for GAPDH mRNA. Significant differences: MSC-treated cells vs. activated cells, **p ≤0.01, ***p ≤0.001

*MSC* mesenchymal stem cells

Flow cytometry studies performed in parallel showed that α4 integrin was constitutively expressed at the plasma membrane level on resting CD3^+^ lymphocytes (mean 52.25 ± 13.87) (Fig. 2c), while very low expression (mean 2.76 ± 1.98) was observed for ICAM-1 (Fig. 2d), in accordance with qPCR data. Upon *in vitro* activation, CD3^+^ cells showed a slight increase in the proportion of α4 integrin (mean 66.81 ± 19.7, *p* <0.01), and a marked increase in the proportion of ICAM-1 (mean 94.5 ± 3.47, *p* <0.001) with respect to resting cells. The presence of MSC in TW slightly reduced the proportion of α4 integrin on the CD3^+^ surface with respect to activated cells (−7 % for 20:1 ratio, −13 % for 10:1 ratio, and −22 % for 4:1 ratio), but the statistical significance was reached only with the highest dose of MSC (*p* <0.001) (Fig. 2c). The presence of MSC markedly reduced also the proportion of ICAM-1 on the cell membrane in a dose-dependent manner (−9.7 % for 10:1 ratio and −15.4 % for 4:1 ratio; *p* <0.05 and *p* <0.001, respectively) (Fig. 2d). No differences were observed for β2 integrin upon exposure to MSC at different ratios (data not shown).

### Inhibition of CXCR3 expression by MSC reduced CXCL10-dependent chemotaxis of T cells

**Fig. 3:**
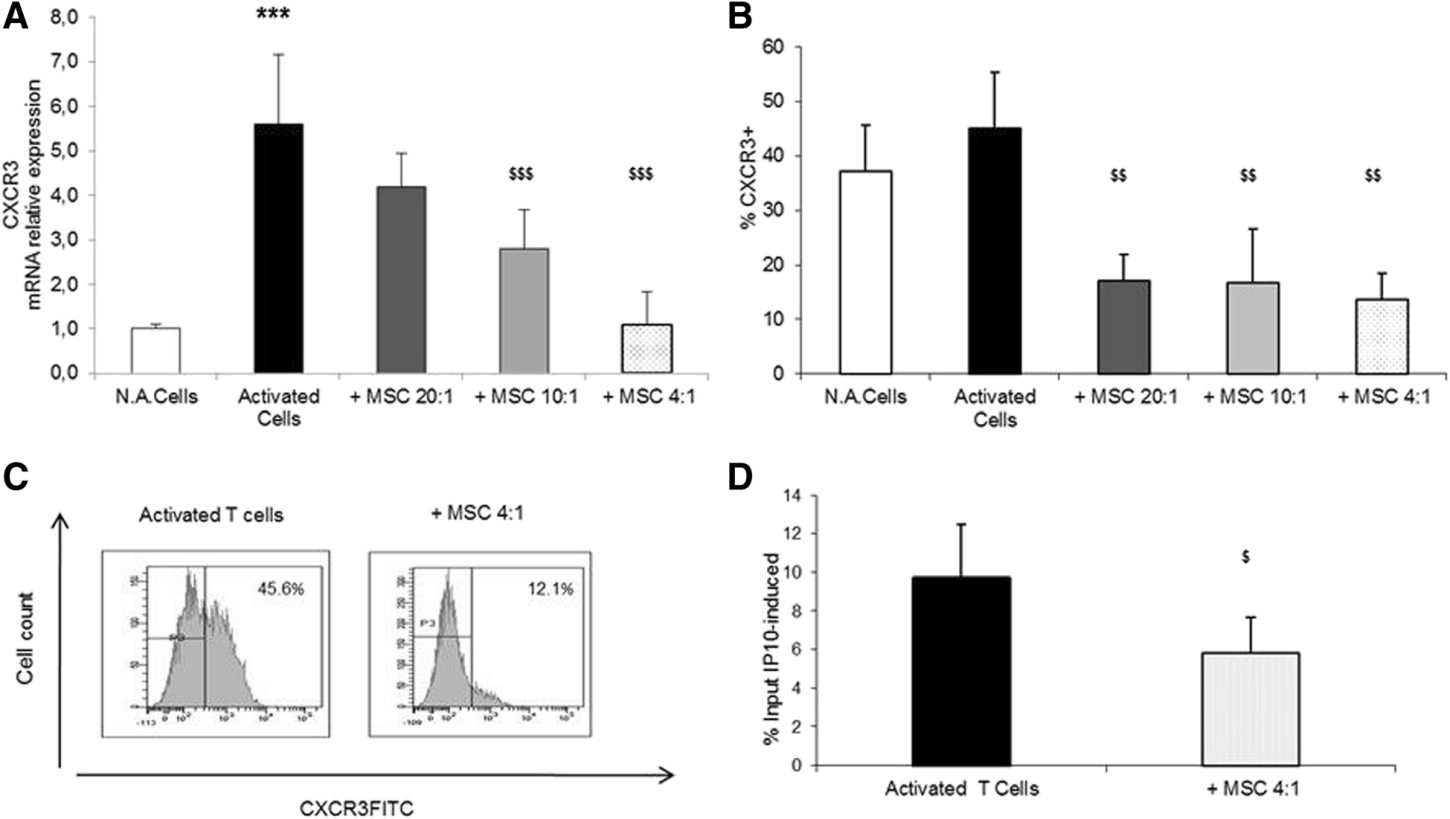
Effects of MSC on CXCR3 expression and function. **a** Activated CD3^+^ lymphocytes were cultured in the absence or in the presence of MSC in TW, and the mRNA expression of CXCR3 was evaluated by qPCR. Data (mean ± SD) are reported as fold induction with respect to controls after normalization for GAPDH mRNA. **b** In the same experimental conditions, the proportion of CXCR3 on T-cell membrane was evaluated by flow cytometry. Results are expressed as mean percentage ± SD obtained from six independent experiments. **c** Fluorescence histograms of CXCR3-labeled CD3^+^ cells cultured in TW for 48 hours with or without MSC (4:1 ratio) in the presence of plate–bound αCD3/αCD28. Results are expressed as median percent of positive cells. **d** Chemotaxis of activated CD3^+^ cells cultured with MSC at 4:1 ratio in response to either CXCL10 or medium alone. Activated vs. N.A. cells, *p ≤0.01, **p ≤0.001, ***p ≤0.0001; MSC-treated vs. MSC-untreated cells, $*p* ≤0.01, $$*p* ≤0.001, $$$*p* ≤0.0001. *MSC* mesenchymal stem cells, *N.A.* PBMC in resting conditions

We observed that CXCR3 was constitutively expressed in resting CD3^+^ cells, and its transcription increased significantly (Fig. 3a) upon activation with αCD3/αCD28 (5.6-fold induction with respect to resting cells; *p* <0.001). This change was still detectable at plasma membrane level (Fig. 3b), although the increase in the CXCR3 proportion on the cell membrane did not reach significance (means 37.1 + 8.58 vs. 45.13 ± 10.21). The upregulation of CXCR3 expression was prevented by the presence of MSC in a dose-dependent manner. Both the 10:1 and 4:1 CD3/MSC ratios were effective on preventing the upregulation of CXCR3, which showed a significant reduction of −50 % and −80 %, respectively, with respect to activated cells (*p* <0.001) (Fig. 3a). These effects were evident also at plasma membrane level, where the presence of MSC led to a dose-dependent reduction in the proportion of CXCR3 on the plasma membrane (−28.1 % for 20:1 ratio, −28.4 % for 10:1 ratio, and −31.5 % for 4:1 ratio; *p* <0.001) (Fig. 3b).

In order to explore whether the effects of MSC reflect on the CXCR3-related T-cell function, the chemotactic activity of activated CD3^+^ cells was assessed. CD3^+^ cells were cultured for 2 days in the absence or in the presence of MSC in TW (CD3/MSC 4:1 ratio). Then, the expression of CXCR3 and the response to CXCL10, the selective ligand of CXCR3, were evaluated. We found a marked decrease in CXCR3 expression on the surface of CD3^+^ cell cultured with MSC with respect to activated cells (means 48.36 ± 10.03 vs. 22.44 ± 11.84; *p* <0.01) that was paralleled by a significant (*p* <0.05) inhibition of CXCL-10-driven chemotaxis (Fig. 3c, d).

### MSC inhibit expression of adhesion molecules on HECV

**Fig. 4:**
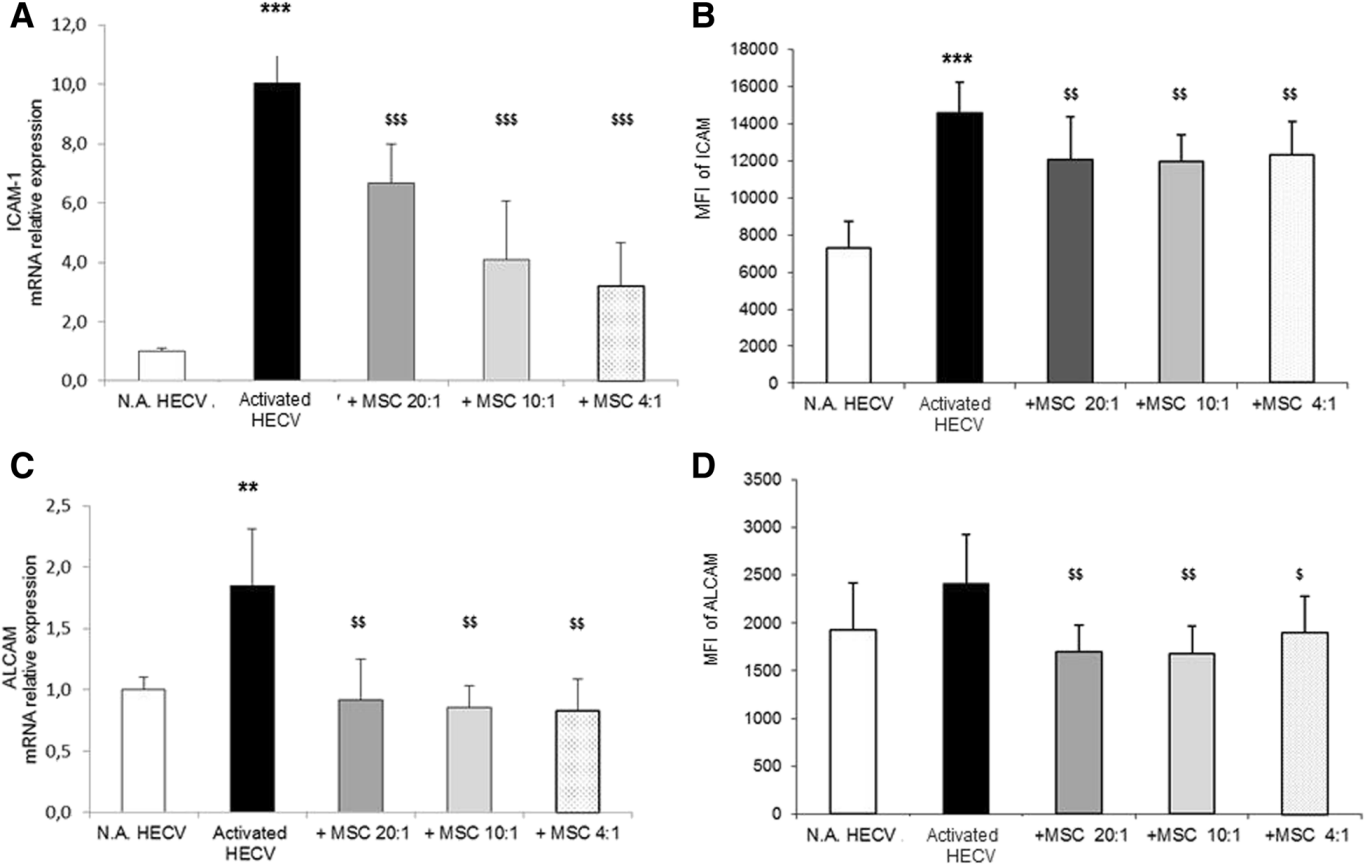
Effects of MSC on expression of HECV adhesion molecules. HECV stimulated using IFNγ were cultured for 24 hours in the absence or in the presence of MSC at the HECV/MSC ratios of 20:1, 10:1, and 4:1 in TW. The relative mRNA expression of ICAM-1 **a** and ALCAM **c** were evaluated by qPCR. Data (mean ± SD) are reported as fold induction with respect to controls. In the same experimental conditions, the mean fluorescence intensity (*MFI*) of plasma membrane expression of ICAM-1 **b** and ALCAM **d** was evaluated by flow cytometry following staining with specific mAb. Results of six independent experiments are expressed as mean percentage ± SD of recorded MFI. Activated vs. N.A. cells, *p ≤0.01, **p ≤0.001, ***p ≤0.0001; MSC-treated vs. MSC-untreated cells, $*p* ≤0.01, $$*p* ≤0.001, $$$*p* ≤0.0001. *ALCAM* activated leucocyte cell adhesion molecule, *HECV* human endothelial cells, *ICAM* intercellular adhesion molecule, *MSC* mesenchymal stem cells, *N.A.* PBMC in resting conditions

The possible effects of MSC on endothelial cells were assessed focusing on some adhesion molecules relevant for their interaction with T cells. HECV constitutively express ALCAM and ICAM-1 at both mRNA and membrane level (MFI = 1927 ± 488.88 and MFI = 7311 + 1398, respectively) (Fig. 4), whereas no constitutive expression of either V-CAM-1 or ICAM-2 was observed both at the mRNA and the membrane level (data not shown).

Upon stimulation with IFNγ, HECV showed a marked increase in ICAM-1 expression at both the mRNA (10.1-fold induction with respect to controls; *p* <0.001) and the plasma membrane (mean 14,599 ± 1635.42, *p* <0.001) levels (Fig. 4a, b, respectively). Also ALCAM expression was upregulated by IFNγ at the mRNA level (1.8-fold induction; *p* <0.01), but the increase was not significant at the membrane level (mean = 2406 ± 520.99) (Fig. 4c, d, respectively). The upregulation of ICAM-1 mRNA expression was reduced by the presence of MSC at all HECV/MSC ratios (−33 %, −59 %, and −68 % for 201:1, 10:1, and 4.1 ratios, respectively, with respect to activated cells; *p* <0.001) (Fig. 4a). Also, at the plasma membrane level, MSC reduced ICAM-1 with respect to stimulated cells (MFI = 14,599 for stimulated HECV vs. MFI = −2517, MFI = −2624, and MFI = − 2288 for 20:1, 10:1, and 4:1 HECV/MSC ratios, respectively; *p* <0.01) (Fig. 4b). The upregulation of ALCAM expression was prevented by the presence of MSC at all HECV/MSC ratios both at the mRNA (about −55 % for all ratios with respect to stimulated cells) (Fig. 4c) and at the plasma membrane level (MFI = 2406 for stimulated HECV vs. MFI = −711, MFI = −726, and MFI = −504 for 20:1, 10:1, and 4.1 HECV/MSC ratios, respectively; *p* <0.01); a slight decrease was observed at the highest HECV/MSC 4:1 ratio; *p* <0.05 (Fig. 4d).

## Discussion

In this study, we demonstrated that MSC significantly act on adhesion molecules and receptors localized on both T-lymphocyte and endothelial cell membrane whose interactions are involved in lymphocyte extravasation and trafficking across the BBB. T-cell homing into the CNS is very low in physiological conditions, but it remarkably increases under inflammatory cues such those occurring in EAE and MS when encephalitogenic CD4^+^ and CD8^+^ cells infiltrate the CNS [1]. The process of leukocyte extravasation consists of a finely regulated sequence of steps that is mediated by different classes of surface molecules sustaining the interaction between leukocytes and endothelial cells [3, 47]. The initial contact of activated lymphocytes with the BBB endothelium is mediated by selectins [3], while integrins trigger the subsequent firm adhesion [11, 12], and then the interaction between ICAM-1 on T cells and ALCAM on endothelial cells led to the final diapedesis [21, 22].

With regards to therapy of MS and other autoimmune diseases, the ultimate goals are twofold: to eliminate/reduce the self-reactive lymphocytes halting the immune attack to the CNS; and to attempt repair of the existing damage. Many studies indicated that transplantation of MSC is of functional benefit in animal models of MS [33, 34]. Different mechanisms have been suggested including induction of immune tolerance toward myelin antigens, release of trophic and anti-apoptotic factors, and induction of endogenous neurogenesis [48]. Interestingly, MSC decrease T-cell infiltration in the CNS of mice with EAE [33] and impair the encephalitogenic potential of T cells [34]. Based on the results presented in this manuscript, we provide a significant insight into the possible molecular mechanism impairing T-cell extravasation under inflammatory conditions as mimicked by the exposure of both T cells and endothelial cells to IFNγ. Although these *in vitro* results should be cautiously considered as validation of the in vivo mechanisms of action of administered MSC and by no means as an indication of optimal yields of cells to be transplanted, they are a solid “proof of concept” permitting to elucidate the possible interactions between MSC, immune cells, and endothelial cells, which might be relevant for some clinical conditions such as MS.

At first, we observed that, *in vitro*, MSC inhibit IFNγ expression by PBMC upon αCD3/αCD28 stimulation using both CC and TW. This clearly indicates that such an effect does not depend on a physical contact between MSC and PBMC, but on soluble factors released by MSC. Moreover, MSC play a profound and dose-dependent inhibition of PBMC proliferation and, interestingly, MSC presence blocks PBMC in G_0_/G_1_ phases of the cell cycle as shown by the decrease in expression of KI67 (data not shown), in accordance with previous reports [28, 49]. On the other hand, MSC exerted the same inibitory effects on selected CD3^+^ lymphocytes.

In the light of their effects on activation of CD3^+^ lymphocytes, we investigated whether MSC act on the pool of molecules involved in T-cell trafficking into the CNS such as α4 integrin, β2 integrin, ICAM-1, and CXCR3. We found a decrease in the level of α4 integrin upon MSC exposure. In particular, we observed that activated T cells overexpressed α4 integrin and this upregulation was prevented by the presence of MSC in TW. This effect of MSC on activated T cells *in vitro* resembles the effects played by the mAb NTZ in vivo [50]. NTZ is “the first of a new class of drugs known as elective adhesion molecule inhibitor” employed to treat relapsing-remitting MS [5], and Harrer et al. [50] described a reduction in both α4 and β1 levels on T-cell, B-cell, and natural killer cell membrane in response to NTZ therapy in MS patients. Our results suggest that MSC may mimic the effect of NTZ on α4 integrin expression in T cells, and this could represent one of the molecular mechanisms supporting the clinical efficacy of MSC transplantation in the animal model of MS. By contrast, β2 integrin, the constituent of LFA-1 antigen required for a broad range of leukocyte actions, was not upregulated in stimulated CD3^+^ cells, in accordance with previous data showing that activation of T cells via the T-cell receptor (TCR) did not change LFA-1 expression [51]. However, MSC are able to reduce the mRNA level of β2 integrin and this action could be synergic with the inhibitory effect on α4 integrin possibly leading to a strengthening of the inhibition of T-cell extravasation.

Activation of CD3^+^ cells also led to an increased expression of CXCR3, a key receptor of Th1 lymphocytes, and this upregulation was inhibited by the presence of MSC in a dose-dependent manner. It has to be noted that T lymphocytes isolated from the cerebrospinal fluid (CSF) of MS patients express high levels of CXCR3 [19] and that CXCR3^+^ T cells are present in the infiltrates observed in MS tissues [19]. Here, we demonstrated that not only expression of CXCR3 on CD3^+^ lymphocytes, but also their ability to migrate under the chemotactic stimulus of CXCL10, which has a relevant role in the pathophysiology of MS [52], is inhibited by MSC. This result is of particular interest because it indicates that MSC also inhibit the effector function of T cells, in contrast to a previous study showing that the inhibitory effect of MSC was confined to proliferation of T cells rather than to their effector functions [53]. Taken together, our data suggest that the inhibition exerted by MSC on CXCR3 expression might cooperate with the other changes induced on het CD3^+^ cell surface to prevent intrathecal accumulation of T cells in MS.

In line with our finding, an earlier report stated that blocking CXCR3 function was associated with less severe EAE and diminished T-cell infiltration of the CNS [54], although other studies reported that CXCR3^−/−^ mice develop more severe EAE [55] and that CXCR3 signaling has a protective role by retaining the T cells in the perivascular compartment [56].

Similar suppressive action of MSC was observed on ICAM-1 expression. ICAM-1 is constitutively expressed at low levels on T cells and is strongly upregulated following stimulation. By contrast, ICAM-1 expression is high in the endothelial cells and is further increased upon stimulation with IFNγ. Here, we demonstrated that MSC modulate ICAM-1 expression on both T cells and endothelial cells by decreasing the frequency of CD3^+^ cells expressing ICAM-1, and the antigen density on HECV. Such an effect is relevant since it indicates a stronger immunomodulation effect of MSC through a bidirectional action that, on one side, blocks ICAM-1 expression on the T-lymphocyte surface and, on the other side, decreases ICAM-1 expression in the endothelial cell membrane. This effect may be considered protective, despite previous controversial results. In fact, some reports showed that blocking of ICAM-1 led to a protective outcome in EAE [4, 57], whereas other studies reported no protection or even increased disease severity [58, 59]. On the other hand, an attenuated disease was observed in ICAM-1 null mutant mice (ICAM-1^null^), which are deficient in all ICAM-1 isoforms [60]. However, we must not forget that blocking of ICAM-1 in vivo affects both leukocyte and endothelial cells, and previous studies showed that the role of ICAM-1 in EAE development was linked to its expression on both endothelial cells and T lymphocytes [61, 62].

When we investigated the possible cross-talk between MSC and endothelial cells, we observed that MSC in coculture with HECV decreased IFNγ-dependent upregulation of ALCAM, an adhesion molecule expressed on endothelial cells [63]. Interestingly, in both MS and EAE, ALCAM expression at the BBB level is upregulated in active lesions [23]. Our finding therefore suggests that suppressive action of MSC on T-cell infiltration is strengthened by their effect on adhesion molecules localized on endothelial cells.

Taken together, our results indicate that MSC act directly on both T lymphocytes and endothelial cells by reducing the expression of those surface molecules highly expressed upon inflammatory stimuli playing a role in the T-cell–endothelial cell interaction at the BBB, thus possibly affecting the consequent lymphocyte entry into CNS. Although these results have been exclusively obtained *in vitro*, the ability of MSC to decrease the expression of key molecules involved in the migration of T cells into the CNS reinforces their therapeutic efficacy in treating MS [48]. Moreover, changes induced on the lymphocyte surface are strengthened by the inhibition of T-cell proliferation and of the chemokine-driven chemotaxis. All of these events may act in synergy and could explain the observation of a reduced number of T cells infiltrating the CNS upon MSC administration [33]. By using different coculture systems we have also clarified that the action of MSC mainly occurs through the release of soluble factors and does not require physical contact between lymphocytes and endothelial cells. Interestingly, these soluble factors are not constitutively secreted by MSC but only under the condition of cross-talk with activated lymphocytes. In fact, when lymphocytes were incubated in MSC-conditioned medium—that is, medium containing soluble factors secreted by MSC in basal conditions without coculture with PBMC—no effects were observed at the level of the surface molecules under analysis (data not shown).

In conclusion, although MSC are described as cells having high differentiation potential, their effector functions seem to be based less on *in situ* transdifferentiation or fusion ability, and more on paracrine effects and cross-talk with other cells present within diseased tissues. In particular, MSC might exert their therapeutic effects also through the inhibition of extravasation of T cells, an event of high relevance for the treatment of MS.

## Conclusions

The results of this study will enhance our knowledge about the mechanisms sustaining the immunosuppressive action of MSC by focusing on the interaction between MSC and endothelial cells. Our findings indicate that the immunosuppressive effect of MSC does not exclusively depend on their anti-proliferative activity on T cells, but also on the impairment of leukocyte migration through the BBB by acting on those adhesion molecules and receptors that are responsible for T-cell rolling.

## Abbreviations

ALCAM: Activated leucocyte cell adhesion molecule
BBB: Blood–brain barrier
CC: cell-to-cell contact
CNS: Central nervous system
CSF: cerebrospinal fluid
DMEM: Dulbecco’s modified Eagle’s medium
EAE: Experimental autoimmune encephalomyelitis
EDTA: Ethylenediamine tetraacetic acid
FCS: Fetal calf serum
GvHD: Graft versus host disease
^3^H-TdR: ^3^H-thymidine
HECV: Human endothelial cell
HSC: Hematopoietic stem cells
ICAM: Intercellular adhesion molecule
IFNγ: Interferon gamma
IgSF: Imunoglobulin superfamily
LFA-1: Lymphocyte function-associated antigen-1
mAB: Monoclonal antibody
MFI: Mean fluorescence intensity
MS: Multiple sclerosis
MSC: Mesenchymal stem cells
NTZ: Natalizumab
PBMC: Peripheral blood mononuclear cells
PBS: Phosphate-buffered saline
qPCR: Quantitative real-time RT-PCR
SD: Standard deviation
TCR: T-cell receptor
Th17: T helper 17 cells
Th1: Type 1 helper cells
TW: Transwell conditions
VLA-4: Very late antigen-4
